# Endothelial, Immunothrombotic, and Inflammatory Biomarkers in the Risk of Mortality in Critically Ill COVID-19 Patients: The Role of Dexamethasone

**DOI:** 10.3390/diagnostics11071249

**Published:** 2021-07-13

**Authors:** Chrysi Keskinidou, Alice G. Vassiliou, Alexandros Zacharis, Edison Jahaj, Parisis Gallos, Ioanna Dimopoulou, Stylianos E. Orfanos, Anastasia Kotanidou

**Affiliations:** 1 GP Livanos and M Simou Laboratories, First Department of Critical Care Medicine & Pulmonary Services, School of Medicine, National and Kapodistrian University of Athens, Evangelismos Hospital, 106 76 Athens, Greece; chrysakes29@gmail.com (C.K.); alvass75@gmail.com (A.G.V.); idimo@otenet.gr (I.D.); 2 First Department of Critical Care Medicine & Pulmonary Services, School of Medicine, National and Kapodistrian University of Athens, Evangelismos Hospital, 106 76 Athens, Greece; alexandroszacharis5@gmail.com (A.Z.); edison.jahaj@gmail.com (E.J.); 3 Computational Biomedicine Laboratory, Department of Digital Systems, University of Piraeus, 185 34 Piraeus, Greece; parisgallos@yahoo.com

**Keywords:** COVID-19, ICU, dexamethasone, mortality, endothelial dysfunction, coagulation, inflammation, suPAR, presepsin, sVCAM-1

## Abstract

Endothelial dysfunction, coagulation and inflammation biomarkers are increasingly emerging as prognostic markers of poor outcomes and mortality in severe and critical COVID-19. However, the effect of dexamethasone has not been investigated on these biomarkers. Hence, we studied potential prognostic biomarkers of mortality in critically ill COVID-19 patients who had either received or not dexamethasone. Biomarker serum levels were measured on intensive care unit (ICU) admission (within 24 h) in 37 dexamethasone-free and 29 COVID-19 patients who had received the first dose (6 mg) of dexamethasone. Receiver operating characteristic (ROC) curves were generated to assess their value in ICU mortality prediction, while Kaplan–Meier analysis was used to explore associations between biomarkers and survival. In the dexamethasone-free COVID-19 ICU patients, non-survivors had considerably higher levels of various endothelial, immunothrombotic and inflammatory biomarkers. In the cohort who had received one dexamethasone dose, non-survivors had higher ICU admission levels of only soluble (s) vascular cell adhesion molecule-1 (VCAM-1), soluble urokinase-type plasminogen activator receptor (suPAR) and presepsin. As determined from the generated ROC curves, sVCAM-1, suPAR and presepsin could still be reliable prognostic ICU mortality biomarkers, following dexamethasone administration (0.7 < AUC < 0.9). Moreover, the Kaplan–Meier survival analysis showed that patients with higher than the median values for sVCAM-1 or suPAR exhibited a greater mortality risk than patients with lower values (Log-Rank test, *p* < 0.01). In our single-center study, sVCAM-1, suPAR and presepsin appear to be valuable prognostic biomarkers in assessing ICU mortality risk in COVID-19 patients, even following dexamethasone administration.

## 1. Introduction

The novel coronavirus causes a wide spectrum of clinical manifestations. A substantial number of patients who will require intensive care unit (ICU) hospitalization will experience severe pneumonia, which may rapidly progress to acute respiratory distress syndrome and multiple organ failure. Data on the prognostic usefulness of biomarkers of endothelial dysfunction, coagulation, and inflammation activation are accumulating. 

Critically ill COVID-19 patients currently receive dexamethasone as a standard therapy [1]. To our knowledge, no study has addressed the prognostic value of biomarkers in patients receiving dexamethasone, nor the effect of dexamethasone on such potential biomarkers.

Hence, we studied the prognostic role of biomarkers of endothelial dysfunction, coagulation, and of the activation of the inflammatory response, in identifying COVID-19 critically ill patients, with a higher mortality risk. To this end, we measured the biomarkers on ICU admission (within 24 h), and associated their levels with worse outcomes.

The purpose of this study was to compare the levels of circulating endothelial dysfunction, coagulation, and inflammation biomarkers in COVID-19 patients, who were treated with dexamethasone in the ICU, with those who had not received dexamethasone. Furthermore, we investigated the prognostic value of these biomarkers on the clinical outcomes of patients.

Our results showed that levels of soluble (s) vascular cell adhesion molecule-1 (VCAM-1), soluble urokinase-type plasminogen activator receptor (suPAR), and presepsin measured in the first 24 h following ICU admission could be useful biomarkers in identifying patients with a poor prognosis, such as mortality, whether or not they had received dexamethasone.

## 2. Materials and Methods

This observational, single-center study included 66 adult Caucasian, consecutive critically ill COVID-19 patients, admitted to the ICU of the “Evangelismos” General Hospital of Athens from 22 March 2020 to 30 October 2020. SARS-CoV-2 infection was diagnosed by real-time reverse transcription PCR (RT-PCR) in nasopharyngeal swabs. The study was approved by the Hospital’s Research Ethics Committee (129/19 March 2020), and all procedures carried out on patients were in compliance with the Helsinki Declaration. Informed written consent was obtained from all patients’ next-of-kin.

Sampling occurred within the first 24 h post ICU admission. Thirty-seven (37) patients from the “first wave” were included who did not receive dexamethasone as part of their treatment during ICU stay; the remaining 29 had received the first dose (6 mg) of dexamethasone prior to sampling, according to the new treatment guidelines [1]. None of the patients were receiving corticosteroids chronically or, prior to the study. Following study enrolment, demographics, comorbidities, symptoms, vital signs, and laboratory data were recorded. Acute physiology and chronic health evaluation (APACHE II) and sequential organ failure assessment (SOFA) scores were calculated on ICU admission. Outcome was defined as overall ICU mortality (it should be noted that the dexamethasone-treated patients underwent the complete dexamethasone regime of 6 mg once daily for up to 10 days during their ICU stay). 

Four milliliters (4 mL) of venous blood were collected within the first 24 h post ICU admission. Serum was drawn in BD Vacutainer^®^ Plus Plastic Serum Tubes (Becton, Dickinson and Company, Franklin Lakes, NJ, USA). Serum was collected, portioned into 0.5 mL aliquots, and stored at −80 °C until used.

The following markers were measured concurrently in samples obtained on ICU admission by enzyme-linked immunosorbent assay (ELISA; the ELISA kits chosen had been previously used and validated in our laboratory): Transmembrane protein 173 (TMEM173) (Wuhan Fine Biotech Co., Ltd., Wuhan, China, intra-assay coefficient of variability (CV) < 8%, detection limit 0.094 ng/mL); triggering receptor expressed on myeloid cells-1 (TREM-1) (R&D Systems Inc., Minneapolis, MN, USA, CV 3.4%, detection limit 15.2 pg/mL); presepsin (Wuhan Fine Biotech Co., CV < 8%, detection limit < 0.094 ng/mL); CD40 ligand (CD40L) (R&D Systems Inc., CV 5.1%, detection limit 10.1 pg/mL); plasminogen (Wuhan Fine Biotech Co., CV < 8%, detection limit < 46.875 pg/mL); plasminogen activator inhibitor-1 (PAI-1) (R&D Systems Inc., CV 6.7%, detection limit 0.142 ng/mL); platelet factor 4 (PF4) (R&D Systems Inc., CV 7%, detection limit 0.1 ng/mL); soluble vascular cell adhesion molecule-1 (sVCAM-1) (R&D Systems Inc., CV 3.1%, detection limit 1.26 ng/mL); soluble platelet endothelial cell adhesion molecule-1 (sPECAM-1) (R&D Systems Inc., CV 3.4%, detection limit 0.075 ng/mL); endothelial cell specific molecule 1 (ESM-1, or endocan) (OriGene Technologies, Inc., Rockville, MD, USA, CV 4.4%, detection limit < 10 pg/mL); ephrin-A1 (Wuhan Fine Biotech Co., CV < 8%, detection limit 0.094 ng/mL); ephrin receptor A2 (EphA2) (Wuhan Fine Biotech Co., CV < 8%, detection limit 46.875 pg/mL); and soluble urokinase-type plasminogen activator receptor (suPAR) (R&D Systems Inc., CV 4.6%, detection limit 33 pg/mL).

Data are presented as individual values, mean ± standard deviation (SD) for normally distributed variables, and median with interquartile range (IQR) for variables with skewed distribution. Two group comparisons were performed by Student’s *t*-test or the non-parametric Mann–Whitney test for skewed data. Associations between qualitative variables were examined by the chi-square test. Correlations were performed by Spearman’s correlation coefficient. Receiver operating characteristic (ROC) curves were plotted using ICU mortality as the classification variable and biomarker levels on ICU admission as prognostic variables. The optimal cut-off value for predicting ICU mortality was calculated as the point with the greatest combined sensitivity and specificity. The Kaplan–Meier method was used for survival probability estimation, and the log-rank test for a two-group comparison. All tests were conducted using a Type I error, α = 0.05 and Type ΙI error β = 0.20 (80% power). The analyses were performed with IBM SPSS statistical package, version 22.0 (IBM Software Group, New York, NY, USA), and GraphPad Prism, version 8.0 (GraphPad Software, San Diego, CA, USA). All *p*-values were calculated after two-sided tests; *p*-values < 0.05 were considered significant.

## 3. Results

**Figure 1:**
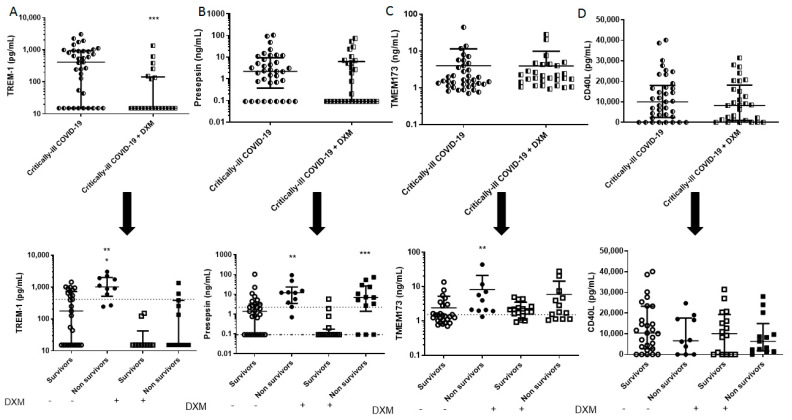
Intensive care unit (ICU) admission levels of inflammatory biomarkers in COVID-19 patients. (**A**) TREM-1, (**B**) Presepsin, (**C**) TMEM173, and (**D**) CD40L were measured in 37 dexamethasone-free and 29 dexamethasone-treated (first dose—6 mg) critically ill patients upon ICU admission (within 24 h) (upper panels). The two patient groups were subsequently categorized as survivors and non-survivors (lower panels). Two-group comparisons were performed with the non-parametric Mann–Whitney test, * *p* < 0.05, ** *p <* 0.01, *** *p <* 0.001. Data are presented as scatter plots, indicating the median value and 25 to 75 centiles. Upper dashed lines, median values of the dexamethasone-free group and lower dashed line, median value of the dexamethasone-treated group. CD40L = CD40 ligand; DXM = Dexamethasone; TMEM173 = Transmembrane protein 173; TREM-1 = Triggering receptor expressed on myeloid cells-1.

**Figure 2:**
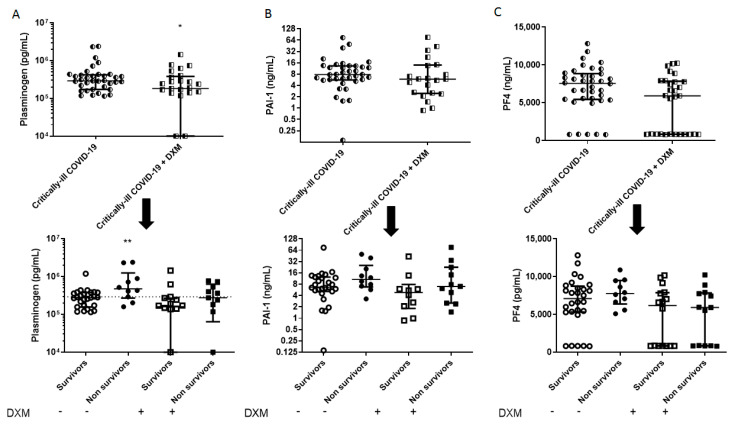
Intensive care unit (ICU) admission levels of coagulation biomarkers in COVID-19 patients. (**A**) Plasminogen, (**B**) PAI-1, and (**C**) PF4 were measured in 37 dexamethasone-free and 29 dexamethasone-treated (first dose—6 mg) critically ill patients upon ICU admission (within 24 h) (upper panels). The two patient groups were subsequently categorized as survivors and non-survivors (lower panels). Two-group comparisons were performed with the non-parametric Mann–Whitney test, * *p <* 0.05, ** *p <* 0.01. Data are presented as scatter plots, indicating the median value and 25 to 75 centiles. Dashed line, median value of the dexamethasone-free group. DXM = Dexamethasone; PAI-1 = Plasminogen activator inhibitor-1; PF4 = Platelet factor 4.

**Figure 3:**
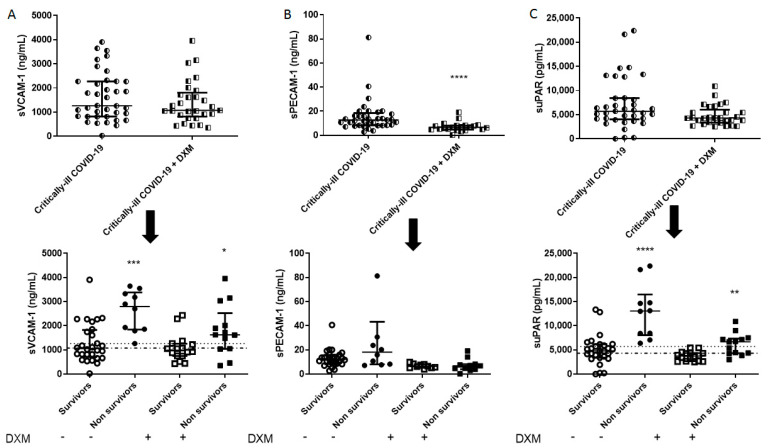
Intensive care unit (ICU) admission levels of endothelial dysfunction biomarkers in COVID-19 patients. (**A**) sVCAM-1, (**B**) sPECAM-1, and (**C**) suPAR were measured in 37 dexamethasone-free and 29 dexamethasone-treated (first dose—6 mg) critically ill patients upon ICU admission (within 24 h) (upper panels). The two patient groups were subsequently categorized as survivors and non-survivors (lower panels). Two-group comparisons were performed with the non-parametric Mann–Whitney test, * *p <* 0.05, ** *p <* 0.01, *** *p <* 0.001, **** *p <* 0.0001. Data are presented as scatter plots, indicating the median value and 25 to 75 centiles. Upper dashed lines—median values of the dexamethasone-free group; and lower dashed lines—median values of the dexamethasone-treated group. DXM = Dexamethasone; sPECAM-1 = soluble platelet endothelial cell adhesion molecule-1; suPAR = soluble urokinase-type plasminogen activator receptor; sVCAM-1 = soluble vascular cell adhesion molecule-1.

**Figure 4:**
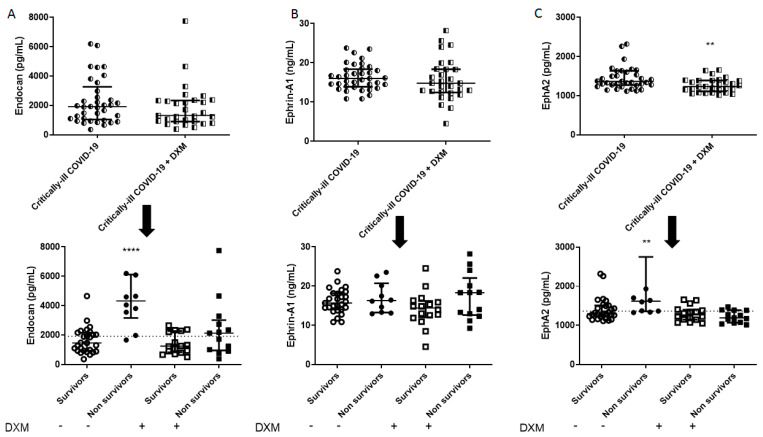
Intensive care unit (ICU) admission levels of endothelial dysfunction biomarkers in COVID-19 patients. (**A**) Endocan, (**B**) Ephrin-A1, and (**C**) EphA2 were measured in 37 dexamethasone-free and 29 dexamethasone-treated (first dose—6 mg) critically ill patients upon ICU admission (within 24 h) (upper panels). The two patient groups were subsequently categorized as survivors and non-survivors (lower panels). Two-group comparisons were performed with the non-parametric Mann–Whitney test, ** *p <* 0.01, **** *p <* 0.0001. Data are presented as scatter plots, indicating the median value and 25 to 75 centiles. Dashed lines, median values of the dexamethasone-free group. DXM = Dexamethasone; EphA2 = Ephrin receptor A2.

The demographics, clinical characteristics and biomarkers of the two patient groups are presented in Table 1. The patients from the “first wave” (March–August 2020), who had not received dexamethasone as part of their treatment, had a higher SOFA score, a lower neutrophil count, and a higher lymphocyte count. Mortality in this group was 27%. As seen in Figure 1, Figure 2, Figure 3 and Figure 4, dexamethasone administration resulted in a decrease in various biomarkers, including TREM-1, plasminogen, sPECAM-1, and ephrin receptor A2. The remaining biomarkers did not seem to be influenced by dexamethasone.

The two groups were subsequently subdivided into survivors and non-survivors, based on overall ICU mortality (Table 2 and Table 3). A seen in Table 2, in the dexamethasone-free group, non-survivors had a higher SOFA score, and white blood cell count. Most importantly, non-survivors had elevated TREM-1, presepsin, TMEM173, plasminogen, sVCAM-1, suPAR, endocan, and ephrin A2 compared to survivors (** *p* < 0.01, *** *p* < 0.001, **** *p* < 0.0001; Figure 1, Figure 2, Figure 3 and Figure 4). TREM-1, TMEM173 and presepsin positively correlated with the white blood cell count, and more specifically, with the neutrophil count (the correlation coefficient r_s_ ranged between 0.4 and 0.5; 0.4 < r_s_ < 0.5, *p* < 0.05). sPECAM-1 positively correlated with the platelet count (r_s_ = 0.35, *p* < 0.05), while suPAR correlated with LDH (r_s_ = 0.56, *p* < 0.0001). sVCAM-1, suPAR, and presepsin correlated with a prolonged ICU stay (0.5 < r_s_ < 0.7, *p* < 0.01).

Alterations in hematological parameters have been shown to be valuable in predicting COVID-19 severity and mortality risk [2]. In line with this, in the dexamethasone-treated group (Table 3), non-survivors were older, had a higher neutrophil count, and lower lymphocyte and platelet counts. Moreover, LDH, D-dimers and CRP were elevated in the non-survivors. Most of the correlations seen in the dexamethasone-free group were lost in the dexamethasone-treated group. More specifically, no correlations were found with cell counts. However, plasminogen correlated with D-dimers (r_s_ = 0.61, *p* < 0.001), suPAR, sVCAM-1, and presepsin with LDH (0.5 < r_s_ < 0.7, *p* < 0.001), while suPAR and presepsin also correlated with CRP (r_s_ = 0.4, *p* < 0.05). None of the biomarkers correlated with a prolonged ICU stay.

Figure 1, Figure 2, Figure 3 and Figure 4 diagrammatically show the levels of all biomarkers studied in both the dexamethasone-free and the dexamethasone-treated group (upper panels), as well as in survivors and non-survivors, respectively (lower panels).

**Figure 5:**
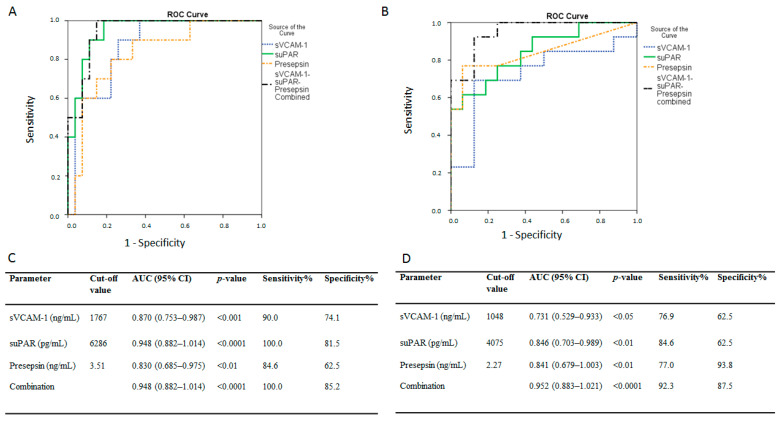
Admission biomarker levels and intensive care unit (ICU) mortality. Receiver operating characteristic (ROC) curve analysis. ROC curves were generated to determine the prognostic accuracy of either suPAR, sVCAM-1, presepsin, or their combination, as measured on ICU admission (within 24 h). ROC curves in (**A**) the dexamethasone-free group and (**B**) the dexamethasone-treated (first dose—6 mg) group. suPAR, solid line; sVCAM-1, dotted line; presepsin, dashed line; combination, dash-dotted line. (**C**,**D**) the corresponding areas under the curve (AUC), 95% confidence intervals (CI) and the optimal cut-off values with the greatest combined sensitivity and specificity are given. suPAR = soluble urokinase-type plasminogen activator receptor; sVCAM-1 = soluble vascular cell adhesion molecule-1.

ROC curves were generated to determine the prognostic accuracy of sVCAM-1, suPAR, presepsin, or their combination, in predicting ICU mortality in the dexamethasone-free group; the areas under the curve (AUC) were: 0.87 (95% CI = 0.75–0.99, *p* < 0.001), 0.95 (0.89–1.01, *p* < 0.0001), 0.83 (0.69–0.98, *p* < 0.01), and 0.95 (0.88–1.01, *p* < 0.0001), respectively (Figure 5A). According to the ROC analysis, the optimal cut-off points, and respective sensitivities and specificities for the biomarkers and their combination are given in Figure 5C. The prognostic accuracy of sVCAM-1, suPAR, presepsin, and their combination, following dexamethasone administration, is shown in Figure 5B; the AUCs were: 0.73 (95% CI = 0.53–0.93, *p* < 0.05), 0.85 (0.70–0.99, *p* < 0.01), 0.84 (0.68–1.00, *p* < 0.01), and 0.95 (0.88–1.02, *p* < 0.0001), respectively (Figure 5B). The optimal cut-off points and respective sensitivities and specificities for the biomarkers and their combination are given in Figure 5D.

**Figure 6:**
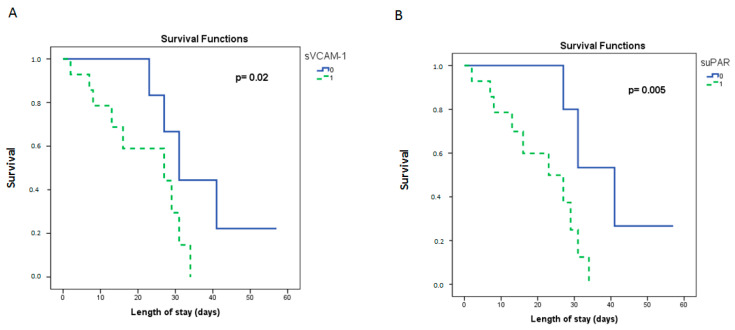
Biomarker levels on admission and intensive care unit (ICU) survival probability. (**A**) sVCAM-1 and (**B**) suPAR were measured on ICU admission (within 24 h). The Kaplan–Meier method was used for ICU survival probability estimation and the log-rank test for two-group comparison. The dexamethasone-treated (first dose—6 mg) group cohort was dichotomized above and below the respective median value of each biomarker. Dashed lines: ≥ median value (high group, 1); solid lines: < median value (low group, 0). The respective median time to mortality for the two aforementioned groups were as follows: (**A**) sVCAM-1, 37 days (95% CI: 26–48) for the low group, and 22 days (95% CI: 15–28) for the high group (Log-rank test, *p* = 0.02), and (**B**) suPAR, 40 days (95% CI: 29–51) for the low group, and 21 days (95% CI: 15–27) for the high group (Log-rank test, *p* = 0.005). suPAR = soluble urokinase-type plasminogen activator receptor; sVCAM-1 = soluble vascular cell adhesion molecule-1.

Kaplan–Meier analysis was next used for survival probability estimation in the dexamethasone-treated group. The group was dichotomized above (high group), and below (low group) the medians of sVCAM-1, suPAR, and presepsin, respectively, as given in Table 1. The probability of mortality with time was significantly elevated in the high sVCAM-1 and suPAR groups (Figure 6A,B). The respective median time to mortality for the aforementioned groups is given in Figure 6.

## 4. Discussion

To the best of our knowledge, this is the first report to study the prognostic value of various endothelial, immunothrombotic, and inflammatory biomarkers in mortality of critically ill COVID-19 patients who were either treated or not with dexamethasone. Our results showed that in our cohort, sVCAM-1, presepsin and suPAR could differentiate patients who did not survive their illness, independently of dexamethasone administration (measured after the first dose—6 mg).

COVID-19 is a progressive disease and, therefore, until now, risk factors, comorbidities, epidemiological data, basic laboratory measurements, and statistical data on the number of cases and deaths have been used in different prediction models [3]. However, given the diversity of the disease, none of the above combinations have been able to provide an early and accurate prognosis. Hence, introducing parameters more specific to the pathobiology of the disease could have a significant impact on prognostic value. Table 4 summarizes the biological function of the biomarkers studied, and previous findings on their role in COVID-19.

Since the regulation of coagulation and thrombolysis, and the promotion of hemofluidity aid in maintaining unobstructed blood flow, targeting these pathways could be a focus of therapy-based approaches in COVID-19 [23]. Furthermore, excessive activation of host innate immune system and coagulation responses can lead to multi-organ failure and death [24]. In our COVID-19 cohort, TMEM173 was not affected by dexamethasone administration. However, in the dexamethasone-free group, TMEM173 was higher in non-survivors, but this was not the case in the dexamethasone group. Another inflammation amplifier, TREM-1, in the dexamethasone-free group was higher in non-survivors on ICU admission; administration of dexamethasone, however, resulted in a significant decrease in TREM-1 levels, rendering it unable to predict mortality. On the other hand, presepsin, an emerging biomarker of infection, especially useful in the prognosis and diagnosis of sepsis [25,26], was not affected by dexamethasone; furthermore, presepsin levels were much higher in non-survivors compared to survivors, with a good prognostic accuracy, as determined from the ROC curves generated. CD40L is also known to have a key role in pathogen infections. In our study, CD40L levels could not be used for the prognosis of poor outcomes in critically ill COVID-19. Our dexamethasone-free non-survivors had higher plasminogen levels compared to survivors. Administration of dexamethasone, however, resulted in decreased levels of plasminogen and loss of its prognostic value. Plasmin(ogen) has been suggested to be useful as an independent factor for risk stratification of patients with COVID-19 [27]. Levels of PAI-1, which is involved in the fibrinolytic process, and PF4, a promoter of blood coagulation, were not indicative of mortality, in our critically ill patients.

The role of endothelial dysfunction in COVID-19 has been established in a plethora of studies. Our results expand on this knowledge. Very recently, it was demonstrated that in COVID-19 patients with moderate-severe respiratory failure, sVCAM-1 levels were higher in non-survivors [18]. The authors suggested that dexamethasone reduces mortality, at least in part, by reversing sVCAM-1-induced endothelial activation and leukocyte recruitment. In our study, one dose of dexamethasone did not seem to reduce the levels of sVCAM-1, and hence, its effects. In both patient groups, whether they had received dexamethasone or not, sVCAM-1 could be assumed as a predictor of mortality, with a good prognostic value. Moreover, as seen in Table 1, in our critically ill cohort, dexamethasone administration did not seem to improve survival. As demonstrated, the benefit on mortality of dexamethasone is observed in COVID-19 patients who are receiving respiratory support, with a clear benefit in those recruited after the first week of their illness, i.e., at the stage when the disease is dominated by immunopathological elements [1]. The discrepancy observed may be due to dexamethasone administration at an earlier stage of the disease. sPECAM-1 levels did not correlate with increased ICU mortality, while non-survivors had higher endocan levels on ICU admission in the dexamethasone-free group; however, in the patients who were treated with dexamethasone, endocan could not predict mortality. Ephrin-A1 and the ephrin receptor A2 (EphA2) are upregulated by inflammatory regulators in injured lungs, suggesting a role in the regulation of endothelial permeability [28]. Our results showed that ICU COVID-19 patients who eventually died had elevated EphA2 levels compared to ICU patients who survived their illness. However, in the dexamethasone-treated group, EphA2 levels were comparable between survivors and non-survivors. Finally, suPAR levels were much higher in non-survivors compared to survivors, whether they had received dexamethasone or not. The biomarker showed a good prognostic accuracy from the ROC curves generated. 

Since endothelial damage was recognized as an important pathobiological mechanism involved in COVID-19, it seemed logical that sepsis/ARDS biomarkers are relative in this disease. In this critically ill COVID-19 moderate-size cohort, out of seven biomarkers that differed between survivors and non-survivors in the dexamethasone-free group, only sVCAM-1, suPAR and presepsin were elevated in non-survivors compared to survivors in the dexamethasone-treated group. Therefore, it seems plausible that reduced mortality following dexamethasone administration may be partly related to the weakened effects of these biomarkers on the activation of the host innate immune system, coagulation responses, or facilitation of the trans-endothelial migration of neutrophils. On the other hand, emerging biomarkers of infection that reflect general activation of the immune system, rather than exerting inflammatory actions, such as suPAR and presepsin, seem unaffected by dexamethasone. The levels of such biomarkers could help with risk stratification management. Indeed, in an open-label trial, early suPAR-guided anakinra decreased severe respiratory failure and restored the pro-/anti-inflammatory balance [29]. 

The limitations of our study must be stated. This study was single-centered, not allowing for generalizations. Multi-center studies are needed to confirm our findings. Additionally, it included a moderate-sized cohort, which did not allow us to perform multivariate regression analysis. 

## 5. Conclusions

Overall, our results point to a link between inflammatory activation and endothelial dysfunction in COVID-19 pathogenesis. sVCAM-1, suPAR, and presepsin could be considered valuable prognostic biomarkers in assessing mortality risk in critically ill COVID-19 patients, even following dexamethasone administration. These biomarkers could be useful in the development of ICU triage/stratification criteria.

## Figures and Tables

**Table 1:** Demographics, clinical characteristics, and biomarkers on ICU admission, and ICU-related parameters.

Characteristics	Dexamethasone-Free Patients	Dexamethasone-Treated Patients	*p*-Value	Reference Values
Number of patients, N	37	29		
Age (years), (mean ± SD)	64 ± 11	63 ± 13	0.9	
Sex, N (%)			0.6	
Male	30 (81.1)	21 (72.4)		
Female	7 (18.9)	8 (27.6)		
Comorbidities, N (%)	25 (67.6)	25 (86.2)	0.09	
Hypertension	17	13		
Diabetes	5	4		
CAD	4	5		
COPD	1	1		
Asthma	1	2		
Hyperlipidemia	9	9		
Hepatitis	1	0		
Symptom days, (mean ± SD)	6 ± 2	7 ± 3	0.3	
BMI (kg/m^2^), (mean ± SD)	26.3 ± 2.3	25.6 ± 2.1	0.2	
APACHE II, (mean ± SD)	15 ± 5	15 ± 3	0.6	
SOFA, (mean ± SD)	7 ± 3	5 ± 2	0.01 *	
PaO_2_/FiO_2_ (mmHg), (median, IQR)	197 (130–250)	129 (97–232)	0.04 *	
White blood cell count (per μL), (median, IQR)	9550 (6225–13,200)	10,145 (6640–11,460)	0.9	4–10.5 × 10^3^
Neutrophils (%), (median, IQR)	82.0 (76.5–87.0)	85.7 (81.5–90.3)	0.03 *	40–70
Lymphocytes (%), (median, IQR)	12.6 (9.0–16.0)	7.9 (5.4–13.3)	0.02 *	25–45
Platelets (per μL), (median, IQR)	219,000 (163,000–270,000)	249,000 (202,000–324,000)	0.3	140–450 × 10^3^
INR, (median, IQR)	1.08 (1.01–1.11)	1.09 (1.03–1.18)	0.5	0.8–1.1
Fibrinogen (mg/dL), (mean ± SD)	633 ± 165	637 ± 170	0.9	200–400
LDH (U/L), (median, IQR)	425 (344–632)	437 (333–585)	0.7	<225
D-dimers (µg/mL), (median, IQR)	0.96 (0.36–2.18)	1.35 (0.78–1.88)	0.3	<0.5
PCT (ng/mL), (median, IQR)	0.73 (0.28–0.93)	0.19 (0.09–1.28)	0.4	<0.05
CRP (mg/dL), (mean ± SD)	13.9 ± 10.2	13.6 ± 9.6	0.9	<0.5
**Biomarkers**				
TREM-1 (pg/mL), (median, IQR)	412 (15–931)	15 (15–143)	<0.001 *	
Presepsin (ng/mL), (median, IQR)	2.2 (0.4–9.4)	0.1 (0.1–6.3)	0.1	
TMEM173 (ng/mL), (median, IQR)	1.5 (1.2–5.8)	1.8 (1.3–4.1)	0.3	
CD40L (pg/mL), (median, IQR)	10,030 (2385–18,015)	8250 (990–18,140)	0.6	
Plasminogen (pg/mL), (median, IQR)	293,000 (172,800–422,400)	182,000 (10,000–383,000)	0.046 *	
PAI-1 (ng/mL), (median, IQR)	7.9 (5.6–13.3)	5.9 (2.5–13.9)	0.2	
PF4 (ng/mL), (median, IQR)	7572 (5442–8856)	5916 (839–7851)	0.05	
sVCAM-1 (ng/mL), (median, IQR)	1264 (818–2274)	1074 (814–1807)	0.4	
sPECAM-1 (ng/mL), (median, IQR)	12.7 (8.3–18.4)	6.7 (4.8–8.1)	<0.0001 *	
suPAR (pg/mL), (median, IQR)	5692 (4035–8423)	4302 (3349–6040)	0.06	
Endocan (pg/mL), (median, IQR)	1924 (1046–3267)	1314 (904–2343)	0.2	
Ephrin A1 (ng/mL), (median, IQR)	16.0 (13.9–18.4)	14.8 (12.4–18.4)	0.3	
EphA2 (pg/mL), (median, IQR)	1366 (1268–1635)	1233 (1112–1635)	0.004 *	
**Outcomes**				
Mortality, N (%)	10 (27)	13 (45)	0.04 *	
LoS in the ICU (days), (median, IQR)	18 (12–39)	17 (9–28)	0.3	
Mechanical ventilation, N (%)	29 (78.4)	22 (75.9)	>0.9	
Duration of mechanical ventilation (days), (median, IQR)	17 (9–38)	11 (7–28)	0.4	

* *p*-value < 0.05. Data are expressed as number of patients (N), percentages of total related variable (%), mean ± SD for normally distributed variables, and median (IQR) for skewed data. For differences between the 2 groups, either Student’s t-test for normally distributed data, the Mann–Whitney test for skewed data, or the chi-square test for nominal data was used. Characteristics and biomarkers were measured on ICU admission (within 24 h). Symptom days refer to the number of days that the patients presented with symptoms, prior to admission. Outcomes were recorded at discharge/death; the dexamethasone-treated patients ultimately received dexamethasone at a dose of 6 mg once daily for up to 10 days. APACHE = Acute physiology and chronic health evaluation; BMI = Body mass index; CAD = Coronary artery disease; CD40L = CD40 ligand; COPD = Chronic obstructive pulmonary disease; CRP = C-reactive protein; EphA2 = Ephrin receptor A2; ICU = Intensive care unit; INR = International normalized ratio; LDH = Lactate dehydrogenase; LoS = Length of stay in ICU; PAI-1 = Plasminogen activator inhibitor 1; PF4 = Platelet factor 4; SOFA = Sequential organ failure assessment; sPECAM-1 = soluble platelet endothelial cell adhesion molecule-1; suPAR = soluble urokinase-type plasminogen activator receptor; sVCAM-1 = soluble vascular cell adhesion molecule-1; TMEM173 = Transmembrane protein 173; TREM-1 = Triggering receptor expressed on myeloid cells-1.

**Table 2:** Demographics, clinical characteristics and biomarkers on ICU admission in the dexamethasone-free group.

Characteristics	Survivors	Non-Survivors	*p*-Value
Number of patients, N	27	10	
Age (years), (mean ± SD)	62 ± 11	68 ± 10	0.2
Sex, N (%)			>0.9
Male	22 (81.5)	8 (80.0)	
Female	5 (18.5)	2 (20.0)	
Comorbidities, N (%)	18 (66.7)	7 (70.0)	>0.9
Symptom days, (mean ± SD)	6 ± 3	7 ± 2	0.8
BMI (kg/m^2^), (mean ± SD)	26.1 ± 2.3	27.1 ± 2.3	0.2
APACHE II, (mean ± SD)	13 ± 5	16 ± 4	0.1
SOFA, (mean ± SD)	6 ± 3	9 ± 2	0.02 *
PaO_2_/FiO_2_ (mmHg), (median, IQR)	202 (139–256)	189 (109–243)	0.4
White blood cell count (per μL), (median, IQR)	8700 (5960–11,080)	13,200 (9338–17,250)	0.03 *
Neutrophils (%), (median, IQR)	79.0 (75.0–86.0)	84.0 (79.0–88.5)	0.08
Lymphocytes (%), (median, IQR)	13.0 (10.0–18.0)	11.5 (6.5–13.8)	0.1
Platelets (per μL), (median, IQR)	207,000 (144,000–258,000)	267,000 (195,000–395,000)	0.08
INR, (median, IQR)	1.08 (1.01–1.10)	1.11 (1.04–1.17)	0.3
Fibrinogen (mg/dL), (mean ± SD)	635 ± 165	627 ± 174	0.9
LDH (U/L), (median, IQR)	418 (335–629)	533 (387–642)	0.4
D-dimers (µg/mL), (median, IQR)	0.76 (0.32–2.23)	1.14 (0.46–2.55)	0.5
CRP (mg/dL), (mean ± SD)	13.1 ± 9.0	16.3 ± 13.1	0.6
**Biomarkers**			
TREM-1 (pg/mL), (median, IQR)	177 (15–726)	1012 (514–1998)	<0.001 *
Presepsin (ng/mL), (median, IQR)	1.4 (0.1–3.2)	12.0 (3.4–23.4)	0.002 *
TMEM173 (ng/mL), (median, IQR)	1.4 (1.0–2.8)	3.0 (2.0–8.2)	0.003 *
CD40L (pg/mL), (median, IQR)	10,300 (2680–23,310)	6520 (6–17,543)	0.6
Plasminogen (pg/mL), (median, IQR)	277,000 (157,000–381,000)	476,000 (271,000–1,300,000)	0.007 *
PAI-1 (ng/mL), (median, IQR)	6.4 (5.2–12.4)	10.7 (6.8–25.4)	0.1
PF4 (ng/mL), (median, IQR)	7094 (5318–8742)	7763 (6380–9492)	0.3
sVCAM-1 (ng/mL), (median, IQR)	1056 (696–1824)	2799 (1840–3383)	<0.001 *
sPECAM-1 (ng/mL), (median, IQR)	12.2 (8.3–15.8)	18.0 (8.1–43.3)	0.2
suPAR (pg/mL), (median, IQR)	4787 (3384–6157)	13,043 (8062–16,471)	<0.0001 *
Endocan (pg/mL), (median, IQR)	1464 (935–2162)	4323 (3161–6116)	<0.0001 *
Ephrin A1 (ng/mL), (median, IQR)	15.7 (14.0–18.2)	16.3 (13.3–20.7)	0.7
EphA2 (pg/mL), (median, IQR)	1300 (1226–1510)	1620 (1359–2750)	0.008 *
**Outcomes**			
LoS in the ICU (days), (median, IQR)	16 (11–31)	29 (17–40)	0.1
Mechanical ventilation, N (%)	19 (70.4)	10 (100.0)	0.08
Duration of mechanical ventilation (days), (median, IQR)	12 (5–37)	26 (17–40)	0.007 *

* *p*-value < 0.05. Data are expressed as number of patients (N), percentages of total related variable (%), mean ± SD for normally distributed variables, and median (IQR) for skewed data. For differences between the 2 groups, either Student’s *t*-test for normally distributed data, the Mann–Whitney test for skewed data, or the chi-square test for nominal data was used. Characteristics and biomarkers were measured on ICU admission (within 24 h). Symptom days refer to the number of days that the patients presented with symptoms, prior to admission. APACHE = Acute physiology and chronic health evaluation; BMI = Body mass index; CD40L = CD40 ligand; CRP = C-reactive protein; EphA2 = Ephrin receptor A2; ICU = Intensive care unit; INR = International normalized ratio; LDH = Lactate dehydrogenase; LoS = Length of stay in ICU; PAI-1 = Plasminogen activator inhibitor 1; PF4 = Platelet factor 4; SOFA = Sequential organ failure assessment; sPECAM-1 = soluble platelet endothelial cell adhesion molecule-1; suPAR = soluble urokinase-type plasminogen activator receptor; sVCAM-1 = soluble vascular cell adhesion molecule-1; TMEM173 = Transmembrane protein 173; TREM-1 = Triggering receptor expressed on myeloid cells-1.

**Table 3:** Demographics, clinical characteristics and biomarkers on ICU admission in the dexamethasone-treated group.

Characteristics	Survivors	Non-Survivors	*p*-Value
Number of patients, N	16	13	
Age (years), (mean ± SD)	58 ± 13	70 ± 10	0.02 *
Sex, N (%)			0.2
Male	10 (62.5)	11 (84.6)	
Female	6 (37.5)	2 (15.4)	
Comorbidities, N (%)	13 (81.3)	12 (92.3)	0.6
Symptom days, (mean ± SD)	8 ± 3	7 ± 3	0.4
BMI (kg/m^2^), (mean ± SD)	26.4 ± 2.2	24.5 ± 1.3	0.01 *
APACHE II, (mean ± SD)	14 ± 3	16 ± 3	0.05
SOFA, (mean ± SD)	5 ± 2	5 ± 2	0.5
PaO_2_/FiO_2_ (mmHg), (median, IQR)	109 (78–142)	196 (127–281)	0.02 *
White blood cell count (per μL), (median, IQR)	9105 (6043–11,300)	11,015 (7263–13,790)	0.3
Neutrophils (%), (median, IQR)	83.6 (80.9–86.9)	90.3 (82.9–93.8)	0.02 *
Lymphocytes (%), (median, IQR)	8.4 (7.8–13.3)	5.3 (3.3–12.3)	0.02 *
Platelets (per μL), (median, IQR)	291,000 (244,000–336,000)	205,000 (185,000–243,000)	0.02 *
INR, (median, IQR)	1.08 (1.03–1.12)	1.12 (1.00–1.34)	0.7
Fibrinogen (mg/dL), (mean ± SD)	604 ± 154	680 ± 186	0.3
LDH (U/L), (median, IQR)	401 (283–501)	603 (390–1183)	0.009 *
D-dimers (µg/mL), (median, IQR)	0.81 (0.41–1.12)	1.28 (0.13–5.21)	0.04 *
CRP (mg/dL), (mean ± SD)	9.2 ± 6.5	19.0 ± 10.1	0.008 *
**Biomarkers**			
TREM-1 (pg/mL), (median, IQR)	15.2 (15.2–42.1)	15.2 (15.2–383.3)	0.1
Presepsin (ng/mL), (median, IQR)	0.1 (0.1–0.2)	6.9 (1.4–26.5)	<0.001 *
TMEM173 (ng/mL), (median, IQR)	2.0 (1.5–2.6)	1.8 (1.1–6.1)	0.6
CD40L (pg/mL), (median, IQR)	10,025 (5.6–19,288)	6270 (1765–14,900)	0.9
Plasminogen (pg/mL), (median, IQR)	160,000 (2535–267,000)	276,000 (64,000–635,000)	0.2
PAI-1 (ng/mL), (median, IQR)	4.8 (1.8–7.9)	7.0 (2.5–22.6)	0.2
PF4 (ng/mL), (median, IQR)	6185 (829–7887)	5916 (850–7841)	0.9
sVCAM-1 (ng/mL), (median, IQR)	985 (744–1254)	1624 (1040–2523)	0.04 *
sPECAM-1 (ng/mL), (median, IQR)	6.4 (5.2–8.2)	6.9 (4.1–8.2)	0.7
suPAR (pg/mL), (median, IQR)	3712 (2668–4341)	6648 (4297–7324)	0.001 *
Endocan (pg/mL), (median, IQR)	1248 (822–2269)	2128 (960–3015)	0.2
Ephrin A1 (ng/mL), (median, IQR)	14.4 (12.1–16.2)	18.3 (12.6–22.1)	0.2
EphA2 (pg/mL), (median, IQR)	1269 (1147–1400)	1192 (1069–1388)	0.4
**Outcomes**			
LoS in the ICU (days), (median, IQR)	14 (8–22)	27 (11–31)	0.2
Mechanical ventilation, N (%)	10 (62.5)	12 (92.3)	0.09
Duration of mechanical ventilation (days), (median, IQR)	8 (5–13)	21 (9–31)	0.008 *

* *p*-value < 0.05. Data are expressed as number of patients (N), percentages of total related variable (%), mean ± SD for normally distributed variables, and median (IQR) for skewed data. For differences between the 2 groups, either Student’s *t*-test for normally distributed data, the Mann–Whitney test for skewed data, or the chi-square test for nominal data was used. Characteristics were measured on ICU admission (within 24 h). Symptom days refer to the number of days that the patients presented with symptoms, prior to admission. Outcomes were recorded at discharge/death; the patients ultimately received dexamethasone at a dose of 6 mg once daily for up to 10 days. APACHE = Acute physiology and chronic health evaluation; BMI = Body mass index; CD40L = CD40 ligand; CRP = C-reactive protein; EphA2 = Ephrin receptor A2; ICU = Intensive care unit; INR = International normalized ratio; LDH = Lactate dehydrogenase; LoS = Length of stay in ICU; PAI-1 = Plasminogen activator inhibitor 1; PF4 = Platelet factor 4; SOFA = Sequential organ failure assessment; sPECAM-1 = soluble platelet endothelial cell adhesion molecule-1; suPAR = soluble urokinase-type plasminogen activator receptor; sVCAM-1 = soluble vascular cell adhesion molecule-1; TMEM173 = Transmembrane protein 173; TREM-1 = Triggering receptor expressed on myeloid cells-1.

**Table 4:** Characteristics of the biomarkers measured in the study cohort.

Biomarker	Biological Function	Studies in COVID-19
TMEM173	Immune adaptor protein that initiates or amplifies inflammation responses, mainly by inducing type I interferon.	No studies on TMEM173. Auto-antibodies against type I interferons are causative of critical COVID-19 [4,5].
TREM-1	Member of the immunoglobulin superfamily, expressed on neutrophils, macrophages and mature monocytes. Acts as an inflammation amplifier, triggering the secretion of pro-inflammatory mediators.	Levels of its soluble form, sTREM-1, in COVID-19 patients could be useful in evaluating the patients’ therapeutic management in the emergency department [6].
Presepsin	Emerging biomarker of infection. Regulates the immune response.	May be useful as a prognostic biomarker for severe COVID-19, and those with prolonged hospitalization [7,8].
CD40L	Member of the tumor necrosis factor (TNF) family. Its soluble form (sCD40L) is mainly secreted from activated platelets.	No difference in sCD40L levels in ICU, non-ICU patients, and non-hospitalized, asymptomatic controls [9].
Plasminogen	Proteolytically breaks down excess fibrin to elevate fibrin degradation products in both bronchoalveolar lavage fluid and plasma.	COVID-19 patients had similar levels as non-COVID-19 sick controls, and ICU-admitted patients had lower values compared to ward patients [10]. ICU COVID-19 patients had lower levels compared to ICU non-COVID-19 patients [11].
PAI-1	Major inhibitor of fibrinolysis, whose upregulation leads to a shift from pro- to anti-fibrinolytic phenotypes.	No difference in ICU, non-ICU patients, and non-hospitalized, asymptomatic controls [9]. PAI-1 levels were elevated in patients with critical COVID-19 infection, and additionally were strongly predictive of in-hospital mortality [12].
PF4	Member of the CXC chemokine family. Exclusively expressed on megakaryocytes and platelets. Enhances inflammatory and coagulant responses and mediates neutrophils degranulation, endorsing their firm adhesion on the endothelium.	Anti-PF4 antibodies are frequently detected in COVID-19 severe patients [13].
sVCAM-1	Member of the cell adhesion molecules. Its primary role is to mediate the adhesion and recruitment of leukocytes to the endothelium during inflammation.	sVCAM-1 levels were elevated in patients with severe disease compared to patients with mild disease and control participants [14,15,16]. In critically ill patients, no differences between survivors and non-survivors [17]. In COVID-19 patients with moderate-severe respiratory failure, sVCAM-1 levels were higher in non-survivors [18].
sPECAM-1	Platelet integral membrane glycoprotein, mainly expressed in endothelial cells, platelets and leukocytes. Comprises an important component in angiogenesis and inflammation, and participates in the trans-endothelial migration of neutrophils.	sPECAM-1 levels have been correlated with disease severity [16,19].
Endocan	Expressed by vascular endothelial cells. It affects leukocytes’ ability to roll and migrate through endothelial cells, but does not disrupt their adhesion.	Higher levels of endocan were associated with higher risk of ICU admission and mortality [20].
Ephrin-A1 and EphA2	Part of the Eph/ephrin receptor-ligand family, known to participate in basic developmental processes, and cell activities that depend on their interaction. They are constitutively expressed in pulmonary vascular cells, and participate in angiogenesis.	-
suPAR	Soluble form of the urokinase plasminogen activator. Plays an important role in the innate host defense in the pulmonary tissue. suPAR levels have been associated with a general activation of the immune system rather than with a particular etiological factor.	Increased plasma suPAR levels in COVID-19 can act as an early predictor of severe respiratory failure [21]. Active suPAR may assist in the early triage of SARS-CoV-2-infected persons to prevent virus transmission [22].

CD40L = CD40 ligand; EphA2 = Ephrin receptor A2; PAI-1 = Plasminogen activator inhibitor 1; PF4 = Platelet factor 4; sPECAM-1 = soluble platelet endothelial cell adhesion molecule-1; suPAR = soluble urokinase-type plasminogen activator receptor; sVCAM-1 = soluble vascular cell adhesion molecule-1; TMEM173 = Transmembrane protein 173; TREM-1 = Triggering receptor expressed on myeloid cells-1.

## Data Availability

Available upon reasonable request.
